# Using human genetic variation to estimate the effect of lipoprotein(a) lowering on pregnancy outcomes

**DOI:** 10.64898/2026.05.18.26351595

**Published:** 2026-05-20

**Authors:** Helena Urquijo, Allison B Goldfine, Juan P Casas, Huilei Xu, Yoav Timsit, Mike Mendelson, Carolina Hache, Ieuan Jones, Daria Arustamian, Maria C Magnus, Tom R Gaunt, Deborah A Lawlor, Maria Carolina Borges

## Abstract

**Background:**

Lipoprotein(a) (Lp[a]) is a genetically determined causal and independent cardiovascular risk factor and Lp(a) targeted therapies are being developed. However, evidence on the safety of substantial Lp(a) lowering during pregnancy is limited. We evaluated the impact of Lp(a) lowering on adverse pregnancy and perinatal outcomes (APPOs) using human genetic evidence.

**Material and Method:**

We applied a drug-target Mendelian randomization (MR) approach using genetic variants associated with Lp(a) in the UK Biobank at the *LPA* locus to proxy pharmacological Lp(a) lowering. Summary-level APPO data were obtained from the MR-PREG collaboration, comprising up to 714,899 women across multiple studies. Twenty APPOs were included. Sensitivity analyses included adjustment for fetal genotype, alternative Lp(a) datasets, leave-one-study-out analyses, and exploration of Lp(a) genetic scores and individuals homozygous for *LPA* loss-of-function variants in the UK Biobank.

**Results:**

Across 20 APPOs, MR estimates showed no strong evidence of causal effects, with no associations surviving false discovery rate *P* -value correction. Most estimates were close to null, including gestational hypertension, gestational diabetes, preeclampsia, miscarriage and neonatal intensive care unit admission. Some associations were slightly larger in magnitude but with wide confidence intervals: gestational age (mean difference 0.04 weeks, 95% CI 0.02–0.06 per 210nmol/L reduction in Lp[a]) and congenital malformation (OR 0.82, 95% CI: 0.72–0.94) in the protective direction of effect, and higher odds of stillbirth (OR 1.09, 95% CI: 1.00– 1.19) and low Apgar at 1 minute (OR 1.11, 95% CI: 0.99–1.24). Sensitivity analyses consistently supported the primary findings, with no evidence of increased maternal nor offspring risk in analyses adjusting for maternal– fetal genotype, across alternative exposure datasets, or in leave-one-study-out tests. Individual-level analyses of Lp(a) genetic score and *LPA* loss-of-function variants showed no associations, although power was limited.

**Conclusion:**

These findings suggest that substantial lowering of Lp(a) is unlikely to increase APPO risk, although modest effects, particularly for rare outcomes, cannot be excluded.

## Full Text Availability

The license terms selected by the author(s) for this preprint version do not permit archiving in PMC. The full text is available from the preprint server.

